# Post-coital intra-cerebral venous hemorrhage in a 78-year-old man with jugular valve incompetence: a case report

**DOI:** 10.1186/1752-1947-4-225

**Published:** 2010-07-26

**Authors:** Beatrice Albano, Carlo Gandolfo, Massimo Del Sette

**Affiliations:** 1 Department of Neuroscience, Ophthalmology and Genetic, University of Genova, Italy, Via De Toni 5, 16132 Genova, Italy; 2 S. Andrea Hospital. La Spezia, Italy

## Abstract

**Introduction:**

Spontaneous intra-cerebral hemorrhage can occur in patients with venous disease due to obstructed venous outflow.

**Case presentation:**

We report the case of a 78-year-old Caucasian man with jugular valve incompetence who experienced an intra-cerebral temporo-occipital hemorrhage following sexual intercourse. He had no other risk factors for an intra-cerebral hemorrhage.

**Conclusions:**

To the best of our knowledge, this is the first case of intra-cerebral hemorrhage due to jugular valve incompetence in association with the physical exertion associated with sexual intercourse.

## Introduction

Non-traumatic spontaneous intra-cerebral hemorrhage is usually due to hypertensive arteriolosclerosis or to amyloid angiopathy, which account for 78 to 88% of primary hemorrhages, whereas secondary intra-cerebral hemorrhage is normally associated with arteriovenous malformation, aneurysm, cavernous angioma, neoplasm, coagulopathy or the misuse of drugs [1].

Ganglionic hemorrhages are probably of hypertensive origin, while lobar hemorrhages are frequently due to amyloid angiopathy or vascular malformations [2].

Some authors have reported the occurrence of intra-cerebral hemorrhages that have been caused by venous diseases. These are usually located in the white matter at the border zone between deep and superficial venous systems where collaterals are poor. Venous intra-cerebral hemorrhages are associated with impaired venous hemodynamics, as in the case of cerebral venous thrombosis, compression of the superior cava vein or right cardiac failure [3]. Moreover, the occurrence of petechial hemorrhages during cerebral venous thrombosis is a frequent finding on computed tomography (CT) or magnetic resonance imaging (MRI) scans [4,5].

We report the case of a patient with bilateral severe jugular valve incompetence in whom a cerebral hemorrhage occurred soon after the physical effort of sexual intercourse.

## Case presentation

A 78-year-old Caucasian man was referred to our stroke unit because of the sudden onset of a headache associated with speech and visual disturbances during early morning sexual intercourse. He had been lying in a supine position with his head hanging off the bed in a slightly downwards position. The patient was brought to the hospital few hours later. On admission, neurological examination showed a right hemianopia with alexia. His systolic blood pressure was 115 mmHg and the diastolic pressure was 60 mmHg.

A thorough review of familial and personal clinical histories suggested no other possible cause for this condition. In particular, there were no signs of arterial hypertension or hematological disorders and our patient was not taking anti-coagulants or anti-platelet drugs. He had not experienced any head trauma and had no other risk factors for cerebrovascular disease. He had also not taken sildenafil citrate or any other cyclic guanosine monophosphate (cGMP) inhibitors.

**Figure 1:**
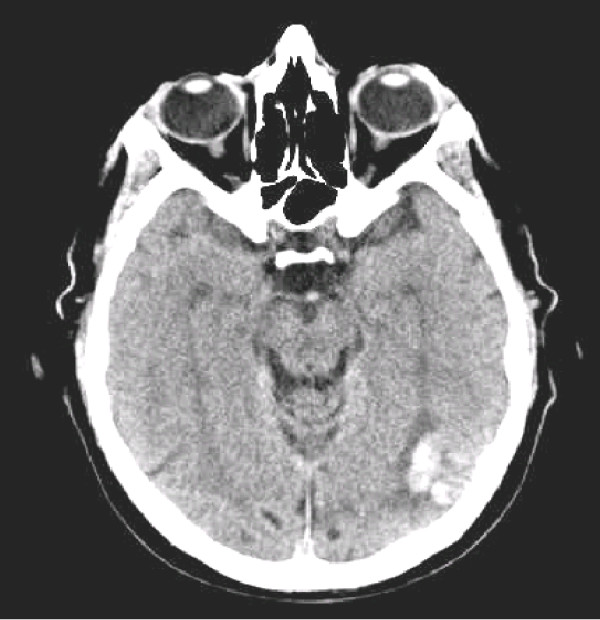
**Cerebral computed tomography scan showing a small left cortical temporo-occipital hemorrhage, with mild mass effect and hypodense halo**.

A cerebral CT scan showed a small left cortical temporo-occipital hemorrhage with mild mass effect and hypodense halo (Figure 1). A carotid and vertebral duplex scan was normal, as was an arterial trans-cranial Doppler. Neuropsychological testing and a neuropsychiatric interview showed no cognitive impairment (Mini-Mental State Examination (MMSE) was 29 out of 30). Routine blood tests, including a platelet count and the plasma level of coagulation factors gave results within normal ranges. A peri-umbilical biopsy for systemic amyloidosis was normal. A cerebral MRI was not carried out because of the presence of abdominal vascular clips. Therefore, a contrast-enhanced CT scan and a traditional digital subtraction angiography (DSA) were performed the following day to rule out the possibility of cerebral venous thrombosis or arteriovenous malformations. These tests provided no evidence of venous thrombosis or vascular malformations, whereas an air contrast ultrasound venography (ACUV) of the jugular veins showed a severe bilateral jugular valve incompetence, with a huge reflow to the brain during a Valsalva maneuver [6,7].

Our patient was discharged a week after presentation, with mild left hemianopia and alexia. The control CT scan showed a partial regression of the cerebral hemorrhage. He was advised to be careful during physical activity and to frequently measure his blood pressure.

## Discussion

We present a case of post-coital intra-cerebral venous hemorrhage in a patient with jugular valve incompetence. We suggest that the physical effort during sexual intercourse (during which our patient's head was hanging off the bed in a slightly downwards position) could have caused the intra-cerebral hemorrhage as there was a close temporal relationship between the physical effort and neurological symptoms.

Post-coital intra-cerebral hemorrhage has been previously described in association with hypertension, the presence of vascular malformations, or the use of sildenafil citrate [8-10].

Although our patient was not hypertensive, the possible contribution of a hypertensive peak during the physical effort cannot be excluded, although in cases described in the literature, such hemorrhages are situated in the deep gray matter. In fact, above the circle of Willis, in the deep gray matter of the basal ganglia and thalamus, arterioles are closer to the direct pulse pressure of the large supplying arteries and there are no branches prior to the arterioles that allow stepwise reduction in pulse pressure [11,12]. In our patient, the lobar cortical hemorrhage was more probably related to local hemodynamic impairment. Lobar hemorrhages are usually due to amyloid angiopathy, vascular malformations, neoplasm, coagulopathies, or the misuse of drugs.

Our patient had no cognitive impairment, no systemic amyloidosis, no neoplasms, vascular malformations or history of drug misuse, so all these possible causes could be excluded.

In reported cases of intra-cerebral hemorrhages due to cerebral venous thrombosis, the pathogenetic mechanism is thought to be the presence of an obstacle to venous outflow represented by a venous thrombus. In our patient, the obstruction to venous flow was caused by the Valsalva maneuver together with the incompetence of the jugular valves, which was 'severe' according to the published classification criteria [13]. The bilateral pathology of the jugular veins did not allow good collateral outflow pathways, and physical effort together with forced expiration are known to be causes of raised cerebral venous pressure [14,15]. Jugular valve incompetence has been reported in association with transient global amnesia and transient monocular blindness [13,16,17], but, to the best of our knowledge, it has not been associated with increased risk of post-coital effort intra-cerebral hemorrhage.

As our patient was not hypertensive, not under any treatment and not affected by any coagulation disorder, and a DSA showed no vascular malformation, we argue that the severe jugular valve incompetence, together with the head-down position, physical effort and forced expiration could have facilitated venous congestion and secondarily, intra-cerebral hemorrhage [14,15].

To the best of our knowledge, this is the first reported case of intra-cerebral hemorrhage during sexual intercourse in a subject with jugular valve incompetence.

## Abbreviations

ACUV: air contrast ultrasound venography; cGMP: cyclic guanosine monophosphate; CT: computer tomography; DSA: digital subtraction angiography; MMSE: Mini-mental State Examination; MRI: magnetic resonance imaging.

## Consent

Written informed consent was obtained from the patient for publication of this case report and any accompanying images. A copy of the written consent is available for review by the Editor-in-Chief of this journal.

## Competing interests

The authors declare that they have no competing interests.

## Authors' contributions

BA analyzed and interpreted the patient's data and collected the literature. CG contributed to writing the manuscript. MDS performed the ultrasound and interpreted the pathophysiology of the cerebral hemorrhage. All authors read and approved the final manuscript.
